# A dominant allele controls development into female mimic male and diminutive female ruffs

**DOI:** 10.1098/rsbl.2013.0653

**Published:** 2013-12-23

**Authors:** David B. Lank, Lindsay L. Farrell, Terry Burke, Theunis Piersma, Susan B. McRae

**Affiliations:** 1 Evolutionary Behavioural Ecology Research Group, Simon Fraser University, Burnaby, British Columbia, CanadaV5A 1S6; 2 Department of Animal and Plant Sciences, University of Sheffield, Sheffield S10 2TN, UK; 3 Chair in Global Flyway Ecology, Animal Ecology Group, Centre for Ecological and Evolutionary Studies, University of Groningen, PO Box 11103, 9700 CC Groningen, The Netherlands; 4 Department of Marine Ecology, Royal Netherlands Institute for Sea Research (NIOZ), PO Box 59, 1790 AB Den Burg, Texel, The Netherlands; 5 Department of Biology and Center for Biodiversity, East Carolina University, Greenville, NC 27858-4353, USA

**Keywords:** polymorphism, alternative male strategies, *Philomachus pugnax*, Mendelian genetics, female mimic

## Abstract

Maintaining polymorphisms for genes with effects of ecological significance may involve conflicting selection in males and females. We present data from a captive population of ruffs (*Philomachus pugnax*) showing that a dominant allele controls development into both small, ‘female mimic’ males (‘faeders’), and a previously undescribed class of small ‘female faeders’. Most male ruffs have elaborate breeding plumage and display behaviour, but 0.5–1.5% are faeders, which lack both. Females from a captive population previously lacking faeders were bred with two founder faeder males and their faeder sons. The faeders’ offspring had a quadrimodal size distribution comprising normal-sized males and females, faeders and atypically small females. By contrast, ornamented males fathered only normal-sized offspring. We conclude that both founding faeders were heterozygous for a faeder allele absent from the original population. This allele is dominant to previously described genes that determine development into independent versus satellite ornamented males. Unlike those genes, the faeder allele is clearly expressed in females. Small body size is a component of the male faeder mating strategy, but provides no obvious benefit to females. Bisexual expression of the gene provides the opportunity to quantify the strength of sexually antagonistic selection on a Mendelian trait.

## 1. Introduction

Alternative mating behaviours and morphs of most species derive from substantial developmental and/or behavioural plasticity, but stable genetic polymorphisms have nonetheless been described in diverse taxa [1–3]. The specific mechanisms maintaining such polymorphisms continue to be debated [1–7], but probably involve the expression of alternative alleles in both sexes [7]. Such selection includes situations with antagonistic selection in males and females, termed ‘intralocus conflict’ [8]. This study documents a locus that provides the opportunity to quantify the strength of this conflict.

The ruff is a Eurasian shorebird with a complex lek mating system that includes a stable trimorphic polymorphism in male mating behaviour and morphology [9–12]. Two morphs are highly ornamented: ‘independent’ males, with dark plumages, defend *ca* 1-m^2^ mating courts against each other on leks. Non-territorial ‘satellite’ males, with white plumages, co-display with independents on courts, while remaining reproductive competitors. A rare third morph (*ca* 1% [12–15]) consists of small, unornamented ‘faeders’ that resemble females, forego male display, and have disproportionately large testes [11,12]. Development into a satellite versus independent is controlled by a Mendelian dominant allele at a single autosomal locus [16,17]. Following the discovery of faeders, we bred them in captivity to determine the mode of inheritance of this third male phenotype.

## 2. Material and methods

We bred ruffs in captivity in 1985–2009. The founders were 56 ornamented males and 64 females hatched from eggs collected near Oulu, Finland in 1985, 1989 and 1990. Two faeders captured during northward migration in The Netherlands [13] were introduced as sires in 2006; they and their faeder sons were bred in 2007–2009.

Ruffs were bred in outdoor aviaries near Kingston, Ontario (1985–1993) and Burnaby, British Columbia (1994–2009). In Kingston, parentage was determined by restricting females’ access to individual males, and monitoring their laying and incubation. In Burnaby, parentage of chicks produced in 2002–2009 was determined using microsatellite markers ([18]; see electronic supplementary material), crosschecked with knowledge of subdivided aviary locations of females and their access to individual males. Chicks were reared in groups organized by hatch date, and subsequent development occurred in common flocks. Culmen and tarsus were measured no earlier than 90 days after hatch, and minimum body mass after six months of age was used as a third measure of body size.

### (a) Morph assignments

**Figure 1.**
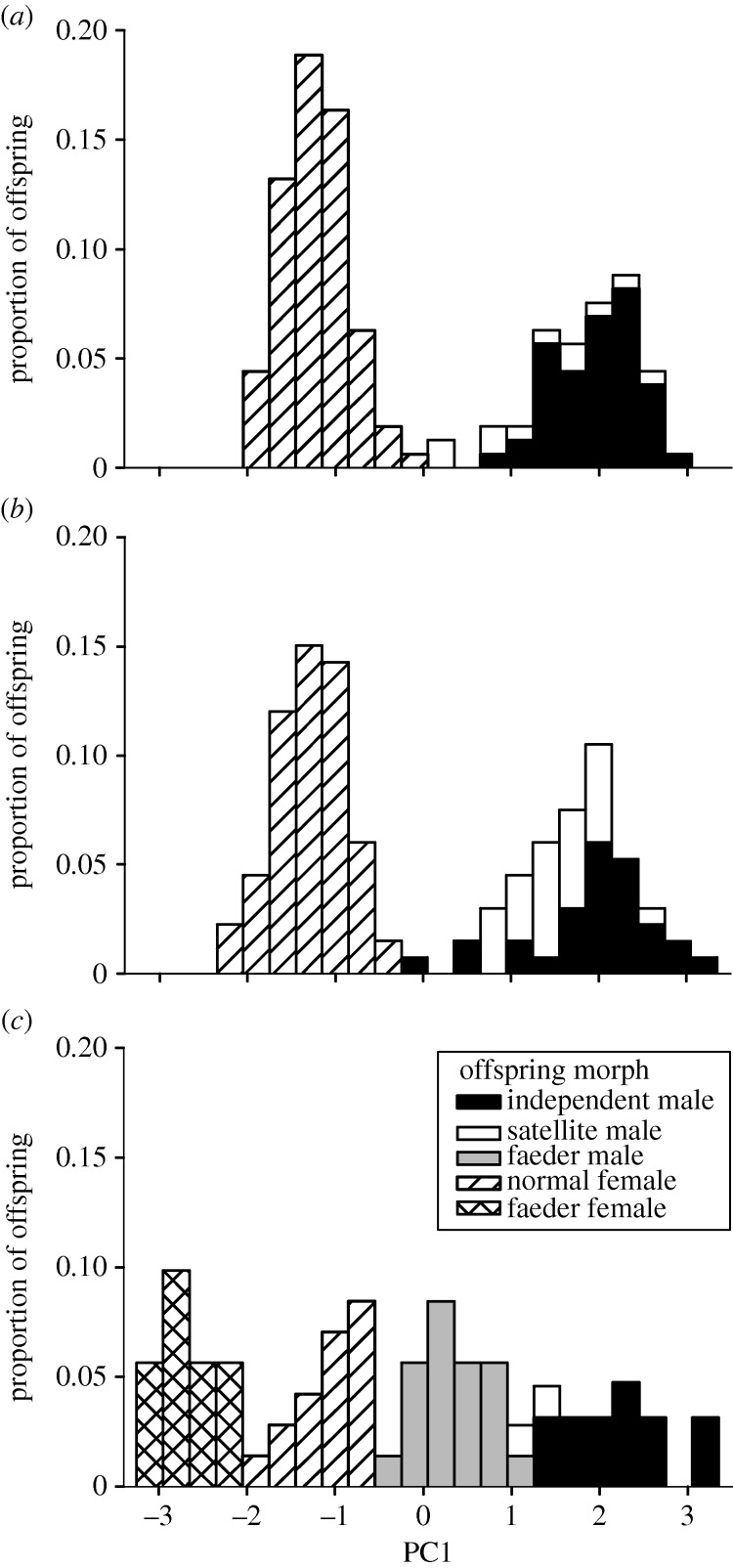
Body-size distributions (PC1, see Material and methods) and (*a*) morphs of the offspring of independent (*n* = 159) and (*b*) satellite (*n* = 133) (both presumed homozygous recessive *ff*), and (*c*) faeder (presumed heterozygous, *Ff, n* = 63) male ruffs mated with females presumed to lack faeder alleles (*ff*). Shading indicates morph type of offspring and sex: males solid, females hatched.

Ornamented males were categorized as independents or satellites based on behavioural observations [17]. Faeders were identified by their lack of breeding plumage and courtship behaviour, and molecular sex determination [19]. As expected from previous studies [11–15], faeders were smaller than ornamented males (table 1 and figure 1). We used logistic regression of known males to assign phenotypes based on body size to males that died prior to expressing a definitive phenotype (see electronic supplementary material). Our analyses are based on birds surviving to fledging, and our interpretations assume no morph-specific biases in prefledging mortality.

**Table 1.** Morphometrics of captive male and female ruff morphs (mean±s.e.). Male morph was determined by behaviour; female morphs assigned based on mode in figure 1.

morph	*n*	bill (mm)	tarsus (mm)	minimum mass (g)
males				
independent	132	35.5 ± 0.1	52.7 ± 0.2	157.6 ± 1.1
satellite	46	34.6 ± 0.2	51.6 ± 0.3	148.0 ± 1.7
faeder	21	32.9 ± 0.3	48.5 ± 0.3	128.6 ± 1.5
females				
normal	246	30.9 ± 0.2	44.7 ± 0.1	90.8 ± 0.4
faeder	19	28.3 ± 0.2	40.4 ± 0.2	73.7 ± 0.9

Following the introduction of breeding faeders, females produced smaller females than had previously been grown in captivity. To try to characterize potential ‘faeder females’, we calculated principal component scores of body size for all males and females, using culmen, tarsus and minimum adult mass. Data were available from 470 ruffs hatched in 1985–2009. PC1 accounted for 0.91 of the total variance, with similar eigenvector weightings for culmen (0.578), bill (0.574) and mass (0.580). The PCA scores allow us to plot the size distribution of all birds on a single scale.

## 3. Results

Faeders were sired exclusively by both founding faeders and four of their faeder sons (table 1 and figure 1). Fifty-five per cent of the 42 sons sired by faeder males were faeders (table 2; 31 different mothers, with one to seven offspring per female, mean = 2.6). By contrast, no faeders occurred among 171 sons of ornamented males produced in 1985–2009 (morph by sire: *LR *χ**^2^ = 88.0, *p* < 0.0001).

**Table 2.** Proportion of faeder offspring sired by faeders mated to females in a population previously lacking faeder characteristics. Daughters were categorized as faeders by their small size (figure 1, see text).

	sons		daughters	
morph of sire	proportion faeder	*n*	proportion faeder	*n*
independent	0.00	91	0.00	144
satellite	0.00	80	0.01	95
faeder	0.55	42	0.48	40
individual faeder sires				
#5474 (wild caught)	0.61	18	0.48	21
#3520 (wild caught)	0.38	8	0.33	6
#302 (son of 3520)	0.60	10	0.38	8
#305 (son of 3520)	0.25	4	0.33	3
#672 (son of 5474)	1.00	2	1.00	1
#308 (son of 5474)	—	0	1.00	1

All of the unusually small females produced following faeder introduction were daughters of faeders (figure 1; tables 1 and 2). We classified as putative ‘faeder females’ 20 birds comprising a mode with the lowest PC1 scores, all but one of which were faeders’ daughters. Assuming that a female raised in 1996 was small for reasons unrelated to faeder genes, we classified her as normal. Based on this boundary criterion, 47.5% of faeders’ daughters were categorized as faeder females (table 2). Female morph class differed by sire (size mode by paternity: *LR *χ**^2^ = 73.6, *p* < 0.0001).

If the faeder trait is determined by a dominant *Faeder* (*F*) allele expressed in both sexes, then we expect half the offspring of heterozygous faeder sires (*Ff*) crossed with homozygous (*ff*) non-faeder females to be faeders (table 2, sexes pooled, GOF **χ**^2^ against expected 1 : 1 morph ratio = 0.00, *p* = 1.0). No other simple genetic model fits these data. In wild populations, male faeders comprise *ca* 0.5–1.5% of all birds [12–15]. Assuming that faeder females occur at similar frequencies to males, and barring assortative mating, the expected probability of faeder × faeder matings is *ca* 10^−4^. It is therefore likely that both founding faeders were heterozygotes.

Since birds have ZW sex determination, and males are homogametic, the *F-*locus cannot be W-linked, but it could be either autosomal or Z-linked. If Z-linked, all female faeder daughters and none of their sons would be faeders, whereas equal proportions are expected in both sexes if the locus is autosomal. These data are unavailable, however, because no faeder female produced offspring during their first potential breeding season, nor did the 2006 or 2007 cohorts do so as 2-year-olds, or the 2006 cohort as 3-year-olds.

## 4. Discussion

A single dominant *Faeder* allele parsimoniously accounts for the inheritance of development into permanent female-mimicking faeder males and a discrete size mode of small females. A previously described autosomal dominant *S* allele controls development into Satellite or Independent male ruffs, with no obvious expression in females (*Satellite* locus [16,17]). *Faeder* could be a super-dominant third allele at the *Satellite* locus, similar to the system determining three male morphs of a marine isopod [5], or be at a separate epistatic *Faeder* locus. As outlined above, if at a separate locus, it may be Z-linked or autosomal. As an alternative approach to determining the genetic architecture, a microsatellite-based linkage map provided no evidence of linkage between markers linked to *Faeder-* and *Satellite-*loci [20]. Thus, epistasis between two autosomal loci appears to account for the inheritance of the three morphs.

In the wild, faeder females should form a discrete mode of *ca* 0.5–1.5% small individuals, parallel to the size mode of faeder males [12–15], unless they are strongly selected against early in life. Six published body-size distributions of migrant ruffs each suggest a very small left-side mode and/or left-skewed tail not previously recognized as being of interest ([12,14,15,21–23], see electronic supplementary material, table S1).

To maintain polymorphisms, alternative alleles must have equal long-term fitnesses [1–5]. Previous considerations of the relative fitnesses of ruff morphs have only considered the mating success of ornamented males [6,10,24,25]. Although a female's *Satellite*-locus genotype can be inferred from male behaviour induced by administration of testosterone [17], there is no obvious reason to expect differential selection on the alternative *Satellite* alleles in females. By contrast, accounting for the maintenance of the faeder polymorphism will require fitness measurements from both sexes [7,8]. Small size is presumably an adaptive component of the faeder males’ ‘female mimic’ mating strategy [12], but may be disadvantageous for females. The young female faeders’ complete lack of production of chicks in the captive flock is unusual. If the *F* allele is strongly disadvantageous for females, we are challenged to understand what limits the evolution of stronger sex-limited expression. Unless other components of fitness offset this apparent fitness disadvantage, faeder males must, on average [3,7], have compensatory fitness advantages over ornamented males, and the system therefore offers an unusually promising opportunity to assess the strength of sexually antagonistic selection on a Mendelian trait [8].

Trimorphic male mating strategy polymorphisms, while uncommon, have been described from several taxa [1,3–5]. A ‘rock–paper–scissors game’, in which each morph achieves higher marginal fitness effects in specific frequency-dependent dyadic combinations, can stabilize persistence [4]. Owing to the rarity of faeders, however, variation in their frequency may not alter the relative mating success of the other two morphs sufficiently for this model to account for stability in ruffs. Gathering data to measure variation in morph fitnesses in the wild will be practical once molecular markers distinguishing morphs become available [20].

## Data accessibility

The data for morph-specific size distributions presented in figure 1 are deposited as electronic supplementary material.
